# Migrations and culture. Essential reflections on wandering human beings

**DOI:** 10.3389/fsoc.2022.1040558

**Published:** 2022-11-25

**Authors:** Paolo Contini, Letizia Carrera

**Affiliations:** University of Bari Aldo Moro, Bari, Italy

**Keywords:** migrations, wandering, boundaries, urban policies, public space, identity

## Abstract

It is possible that our century and the one just past will be remembered in the future as the centuries of migration. Faced with the extent of migration today, social scientists have posed several questions, and in particular they have examined the causes of migration, the integration of immigrants into host countries, and the development of their cultural identity. The newcomers are almost always poorer than those who settled before them, and have different languages, physical appearance, customs, beliefs, and religious practices. The widespread perception is that of an upheaval of the social order. For some, it is the dawn of a new world, under the banner of métissage (or hybridization) and universal brotherhood; for most, it is the beginning of an invasion. Immigration is always a matter of borders: Who is “us”? Who is “them”? The host society has the power to define, classify, and construct the social category of immigrants. There are many differences within this category and obviously any strategic policy should be able to manage every specificity. Nevertheless, we need starting by focusing the more general ideal type of what is “otherness” and what it can be in the social representations in order to construct new conditions of encounter. In this scenario, urban spaces represent the stages on which the encounter with difference takes place. The space is never neutral and may affect, sometimes significantly, the conditions of that encounter. The physical form of the city is the result of widespread social representations of all phenomena, but it is also able to act on those same social representations by altering the processes that take shape within it. The urban dimension and the redesign of the urban space become increasingly key to shaping and managing social processes aimed at governing the transformation processes in a multi-ethnic sense of the European societies. Urban policy-makers can either wait for spontaneous processes of integration and virtuous composition of differences, or implement actions to manage differences, to prevent potential conflicts and start processes of active inclusion. In order to support the act of wandering within this second pattern of urban policies, moving from simple tolerance to a Habermasian process of dialogical exchange, at least two conditions are necessary: the existence of shared public spaces, and the quality of policies for regulating the use of urban public spaces.

## Introduction

It is possible that our century and the one just past will be remembered in the future as the centuries of migration. Despite being a constant feature in history, migration has become increasingly common thanks to the development of transport and communication networks. While leaving long-term evaluations to future historians, we believe that certain attempts by social scientists to understand and address a phenomenon of epochal significance may be useful. Faced with the extent of migration today, social scientists have posed several questions, and in particular they have examined the causes of migration, the integration of immigrants into host countries, and the development of their cultural identity (Laplantine and Nouss, 1997). If it is true that we all have African origins, then migration is as old as humanity itself. Archaeological research, Homeric poems, and biblical accounts tell us about the movements of individuals and groups, commercial exchanges, peaceful colonisations, and bloody invasions: “movement” experiences that have built the history of human civilizations. Permanence, achieved with considerable effort in the Neolithic, has never been absolute: the movement of populations, in various forms and with different outcomes, has always played a role in the formation of stable societies.

Today, once again and with a disruptive impact, migration is one of the most visible and controversial factors of change in our societies. In urban spaces, in the job market, in classrooms, in places of religious meeting, in the world of illegal activities, replacements, and mixes of old and new protagonists are ongoing. The newcomers are almost always poorer than those who settled before them, and have different languages, physical appearance, customs, beliefs, and religious practices. The widespread perception is that of an upheaval of the social order. For some, it is the dawn of a new world, under the banner of métissage^1^ (or hybridization) and universal brotherhood; for most, it is the beginning of an invasion. Overall, in 2020, migrants represented approximately 3.6% of the world population: around 281 million out of over 6 billion human beings^2^, while in the EU-28 area, there were 39.9 million migrants, an increase of 3.5% compared to 2017. As regards distribution across the various countries, over 9 million foreign residents were hosted in Germany, Italy, France, Spain, and the United Kingdom. On January 1, 2020, the population of Italy was 60,244,639, of which 5,506,348 were foreign citizens (9.1%) and 2,748,476 were women (51.8%).

In percentage terms, the numbers are small, but perceptions do not reflect the data, and this is because aspects such as concentration of migrants in certain target areas, the rapidity of the formation of new flows, and the dramatic nature of the arrival of many migrants increase the sense of bewilderment and threat.

## “Us” and “Them”: The sense of boundaries

Who are the immigrants? This is the first issue: it is not easy to define who the immigrants are, or more precisely, who should be classified as such. Immigration is always a matter of borders: who is “us”? Who is “them”? By “us” we usually mean not only the natives but also “our friends,” that is, foreigners we welcome as residents and, possibly, as future fellow citizens; by “them” we mean strangers who we are willing to admit provisionally but who, in principle, we would never want to see settled in our cities, and least of all as full citizens or neighbors.

The host society has the power to define, classify, and construct the social category of immigrants, in the sense of foreigners from poorer countries who are allowed to stay provisionally and conditionally: both norms and common sense, as well as language, contribute to the delimitation of social boundaries where immigrants are concerned. We usually define as “immigrants” only some of the foreigners who reside permanently and work in our country: the British or the French are not “immigrants,” and neither are the Japanese or Koreans, even when they fall within the conventional definition of immigrant adopted by the UN:

“Any person who is moving or has moved across an international border or within a State away from his/her habitual place of residence and who has lived in that country for more than a year.”

The same applies to the term “non-EU,” a legal concept that has become almost synonymous with “immigrant,” with paradoxical consequences: for example, it does not apply to Americans, but it does apply to Romanians. Immigrants are therefore people from poor countries or countries whose culture is perceived as significantly different from ours. Simply (and brutally) put, we can argue that the definition of immigrant is closely linked to our mental boundaries, and these boundaries seem to be unstable and irregular. A black person is perceived as an immigrant, a rich Arab is perceived as a friend: as argued by Ambrosini “wealth can whiten the skin” (Ambrosini and Abbatecola, 2009, p. 13).

Immigration is, therefore, not only a matter of population movement but a much more complex issue, in which the policies of the host countries have a huge impact on the (implicit or explicit) ways in which foreigners are categorized, as well as on the way in which society reacts to new arrivals, and, of course, on the migrants themselves.

Therefore, while, on the practical level, the phenomenon is “simply” constituted by movement from the “poor” side to the “rich” side of an incredibly unequal world, from a cultural point of view the implications are profound and multiple.

It is worth highlighting the fact that the migratory phenomenon often has heavy repercussions both on the countries of origin and on the host countries: migration and immigration, which are complementary in the sociology of Sayad (2002), together make up a unique phenomenon, as every immigration into a society always corresponds to an emigration from another society. Relations between human beings are transformed by migrations, by the arrival and settlement of migrants (but in the countries of departure, relations are transformed by the departures and prolonged absences of the migrants themselves), affecting the cohabitation and coexistence of social groups and individuals in a given territory and its communities. The countries of origin (the “poor side”) see their human capital, both actual and potential, depleted, since migrants are often young people who are a key part of the workforce and the (potential) intellectual resources: the so-called “brain drain” is a further disastrous consequence on the already poor countries of origin.

## The complexity of a “total social fact”

Sayad's analysis is still perfectly relevant to present-day migration: Sayad studied Algerian migration to France in the 1970s, a mass migration from a predominantly rural society to an urban and industrial society (Sayad, 1976); now, as then, the migratory phenomenon can be seen as a direct consequence of colonization; now, as then, it has as its landing places countries that represent extremely rigid models of nation-states, representatives of that “universal imperialism” mentioned by Pierre Bourdieu (1984, 1998, p. 85). In addition to a huge social and economic commitment, host countries are required to carry out a cultural conversion that is anything but superficial, which may involve the renegotiation of shared meanings and of the sometimes profound traits of collective identity. Cultural pluralism is essential to the avoidance of ethnocentrism and all forms of discrimination, and as a means of encouraging an ethic of recognition and respect for differences. Cultural pluralism is a broad paradigm that “contains” at least three perspectives: interculturalist (Hannerz, 1996, p. 36), multiculturalist, and transculturalist (Welsch, 1999).

The interculturalist perspective focused largely on the topics of diversity and otherness: where emphasis is placed on the differences between groups, there is a risk of creating even more distance between them and initiating, even if unwittingly, segregational processes, and ghettoization. Cultural barriers are thus maintained and stereotypes are reaffirmed and reinforced. The process of recognition and enhancement of otherness can lead to pointless and often harmful essentialism and to excessive idealization, by minorities, of the culture or country of origin (the idea of authenticity, produced by the nostalgia of “pure origins,” is also a consequence of this phenomenon that needs to be re-evaluated and superseded). Therefore, despite good intentions, the intercultural dream can be counterproductive and can lead to the exacerbation rather than the resolution of cultural conflicts. Going beyond the difficulties deriving from the dissemination of the intercultural project, it is worth asking whether we should proceed today, as Demetrio suggests, to “a variation of the paradigm, a different conception of culture and relations between cultures” (Demetrio, 2003, 177).

Currently, given its evident inadequacy to explain the complexity of today's phenomena, the traditional notion of “culture” needs to be in some way revised. In the social, anthropological, and psychological sciences in particular, we refer ever more often to transculturality and transculturalism. These new concepts place emphasis on the dialogic character of cultural influences, and tend to a conceptualization of interaction in which nothing is completely “other” (foreign and extraneous); they are a useful way to understand the processes of formation of the multiple identity of the subject (both as an individual and as a community) in all its complexity.

The issue that needs to be addressed is related to the idea of “culture” that should underlie a pluralist society, especially an intercultural one: if this issue is not resolved, aporias, and paradoxes may emerge. The enlightened West has identified its modern utopia in the concept of multi-culturalism: multicultural society is (or should be) welcoming toward otherness, willing to “embrace” differences. Multiculturalism and its direct offshoot, interculturality, have proved to be weak because they are anchored to an idea of culture which is characterized by social homogenization, ethnic consolidation, and intercultural delimitation (Welsch, 1999, p. 194), while the real experience, the increasingly dense and complex cultural interconnections of the process of globalization and transnationalization show all the fragmentation of the social mosaic, and reveal the inadequacy of a closed conception of cultural systems, which have always been nourished by and lived on hybridizations and exchanges. The notion of transculturation emerged as early as the 1940s, when it became necessary to broaden the concepts of acculturation and deculturation (Ortiz, 1940). Today in cultural studies, especially in the colonial and postcolonial fields, the term transculturation has gone beyond its original unidirectional meaning and has come to signify reciprocal interaction (Pratt, 1992, 1995): it is, therefore, legitimate to see in transculturation, in the sense of a model of multidirectional cultural exchange, a forerunner of today's concepts of transculturality and transculturalism. Modern reality, which is profoundly and obviously affected by transnationalization (an expression that anthropologists prefer to the more generic globalization), therefore requires a sort of “fine-tuning” of the notion of culture, mainly from the point of view of flexibility. The endless “contaminations” in the economic, financial, and political spheres, which are the basis of the modus operandi of late capitalism have led to a continuous alteration of cultural meanings and identities: transnationalization has not produced the homogenization of culture, or the creation of a “diffuse indistinct”; rather, with the variety of its collateral phenomena (migrations, circulation of knowledge, ideas, products), it is characterized by an evident increase in cultural diversity, a new form of diversity, resulting from the dense interconnections and growing deterritorialization that make it increasingly difficult to pigeonhole different cultures as “discrete units.”

Particular articulations of the concepts of global and local in modern societies give rise to new cultural forms, both modern and plural. In order to explain the processes of formation of “migrant modernities” (Schulze-Engler, 2002) and virtual community identities, which are localized cultural expressions produced by globalization (Appadurai, 1996), it is necessary to develop new conceptualizations and models of cultural interaction. The concept of transculturality developed by Welsch is operational as well as descriptive and, Nietzsche being a precursor, the emphasis is placed on cultural fertilization at several levels, from the macro level of societies—whose cultural forms are increasingly characterized today by internal differentiation, complexity, and hybridization—to the micro level of individual experience, where personal and cultural identity almost never corresponds to civic and national identity but is instead ever more clearly characterized by multiple cultural connections. The transculturalist model is, at least on a theoretical level, a hypothesis that can be pursued both globally and locally; it is the “third way,” an alternative to other models that have been presented (more or less covertly) as assimilationist. To overcome the temptation to do the same, we find ourselves obliged to re-examine the question of borders, not in a political sense, but rather from the cultural and ideological point of view.

As a prelude to trying to break down barriers, it is worth thinking about the topics of culture and diversity. We tend to consider “cultural diversity” as a sort of conceptual sphere within which there are differences linked to religious denominations and geographical origins: this type of approach is often taken to extremes and tends to be associated with every immigrant, or with every group of immigrants; it is based on the idea that there is a “culture” that everyone carries with them, as if it was a static dead weight. This is considered either a problem or a resource, depending on the way in which the culture of the country of origin is perceived. This perspective, which seems to be dominant, has a limit, which is linked to the static nature that is attributed to culture, and to the fact that culture is not thought of, as it should be, as a process that can change, that evolves in close connection with the context. It is evident that the culture of every social group, of any size, includes among other things, nations, ethnic groups, cities, neighborhoods, work organizations, gender, and generational groups (Barrett, 2013): from this perspective we can observe how each can belong simultaneously to more than one culture, and we might even conclude that, although they occupy the same space, not all of these cultures coexist harmoniously. The diversity of the world is infinite, and cultural diversity has always existed as a human condition (De Sousa Santos, 2011), which today, in a globalized world characterized by the phenomenon of migration that may now be legitimately defined as a “total social fact” (Sayad, 2002), is clearly visible.

## Near and far: From the reflection on immigration to the reflection on migration

The modes of relationship with respect to diversity or, to use Simmel's words, to the other and to otherness, in the sociological conceptualization of Simmel's own interpretative categories of distance and proximity, difference and similitude, configure contrasting feelings specific to these relationships. The foreigner, although belonging to the community in which he is located, is defined by this relationship of distance and proximity, which gives rise to mechanisms of acceptance or rejection. The ambivalence of the sociological category of the foreigner, in the relationship that is established with the other, is therefore the bearer of a change thus drives change in the consolidated social space.

Social sciences, starting from the issues related to the settlement of migrants, initially examined migration with respect to the immigration economy and the process of social and national integration (Rea and Tripier, 2003). Anglo-Saxon social anthropology has focused primarily on the notion of social networks (Hifly et al., 2004), and the transnational dimension of such networks, highlighted by the French, has led to an understanding of a transnational form of migration, that of itinerant communities^3^. The relevance and characteristics of contemporary migrations therefore require us to grasp the modes of “collective existence” of migrants and the subjective dimension of the experience of itinerant communities, where individuals are united by their journey to Europe and by a changing transmigration that ends up favoring métissage and soliciting multiple systems of belonging. From being a marginally researched topic in the human and social sciences, as pointed out by the aforementioned Abdelmalek Sayad, migration has now become a fully-fledged fundamental research subject and means of questioning the social bond and the relationship with otherness, where the others bring with them social stories that must be “re-cognized,” “re-elaborated,” and examined together with the features that characterize them: traditions and religions, as well as social, political, and mental aspects^4^.

The wandering of migrants, the transmigration of people and social stories between worlds and cultures, involves changes and is at the same time a reciprocal fecundation of diversities that are mutually enriched (Le Quéau, 2007). However, the act of wandering requires a common space (Cambi, 2006) to be shared by different sensitivities and cultural heritages, and this is in part denied them by a society that is afraid of otherness, where the encounter becomes the narration of more than one otherness (Tarsia, 2010). The theme and experience of the narrative, which has become a significant element of contemporary culture and social practice in which various people share a story, represent a fundamental approach to the relationship with different cultures (Della Porta et al., 2000; Jedlowski, 2000; Melucci, 2000). While social research cannot neglect and ignore people's social story, at the same time understanding of human relationships needs to be based on observation of the spaces through which people pass and live, and the relationship with ourselves and with the other needs to be considered in the context of the relationship with the living space. All these elements are what the broader concept of culture is “made up of.”

## Cities as places of identity and cultural conflict

Urban spaces are the stages on which the encounter with difference, in the sense Goffman intended, takes place. Nevertheless, these spaces are never neutral and may affect, sometimes significantly, the conditions of that encounter. The physical form of the city is the result of widespread social representations of all phenomena, but it is also able to act on those same social representations by altering the processes that take shape within it. As Henri Lefebvre wrote, the city is, in fact, a sort of synecdoche that gives physical form to the processes that characterize the whole society. Georg Simmel had already understood its heuristic potential when he wrote “the city is not a spatial fact with sociological effects, but rather a sociological fact that is spatially formed” (Simmel, 1903, tr.it. 2018, p. 756). Analysis of the city is an essential starting point that allows us to look at society and the phenomena that characterize it in a place where those phenomena are highly visible. As Michel De Certeau observed, social forms reveal themselves in the *deus absconditus* that inhabits the cities. He wrote “the city gives shape to society.” At the same time, the space and the spaces of the city create the conditions, and even the same possibility, for the occurrence of processes, connecting to them in a logic of active and circular reciprocity.

Immigrations, which are by definition a global phenomenon, also take on a specifically urban character, which cannot be ignored both in terms of understanding this phenomenon and in terms of policies and repercussions on daily life. Within the framework of migrations the city-society link becomes particularly marked and essential. The traits of diversity and extraneousness are constitutively those of the metropolitan ideal type described by Simmel (1903) and by Burgess (1925). Starting from these traits, the city is the place of differences, and within it, as Simmel wrote, “everyone is a foreigner.”

After a century, in the current *reflexive modernity* (Beck et al., 1994), the cities have radicalized the features of the metropolitan ideal type; they are increasingly anonymous, dense, and inhabited by strangers. Everyone in that new urban space is increasingly foreign because there are no conditions for recognition. The more and more cities have experienced a multiplication of differences and of the complexity linked to managing them by policies able to internalize the complexity itself. Citizens are obliged to deal with the idea of difference and of multiplicity, to live closer to others who are also deeply different, even those who, as Lidia Ravera observed, bear the signs of that difference “on their skin.” This characteristic is now part of the human geography of cities, including those that are medium-sized or small.

Until the last part of the twentieth century, the modern-industrial city could be deciphered and described on the basis of differences and spatially identifiable boundaries and was based on the overlapping of social distance and territorial distance, which were kept constant by the market and public policies. The deep contradiction experienced by increasingly fractured cities is actually “a structural characteristic, not an incidental or residual one, of the advanced phases of capitalist accumulation, which emerge in various forms in different space-time contexts and substantially create imbalances and conflicts between elites and poor masses” (Pezzano, 2020, 33). In the last century, as Castells (2002, 2003) pointed out, due to the huge increase in social inequalities, socio-spatial injustices, originating from global capitalism, have emerged^5^. In addition, still today in large western cities there is a constant attempt to make rich and poor people increasingly spatially distant and every social injustice has its own translation in spatial terms (Secchi, 2013). “The space is the objectification of the social (…) It is a political instrument deliberately manipulated” (Lefebvre, 1976, p. 40, 41). Of course the new poverties go also beyond racial issues, but the immigrant status continues having a significant importance in defining the quality of life of the different subjects who end up living parallel lives within the same urban space, are dramatically central.

As we have already noted, the condition of immigrant is not a monolithic, but it presents a deep differentiation. Urban policies and, before them, national and international ones should confront themselves and manage this complexity based on ethnicity, gender, religion, culture, age, and so on, in order to recognize different conditions and to develop effective interventions and strategies.

Talking about immigrants is based on this awareness. At the same time, each reflection should start from facing the ideal type of “the other” in order to articulate more complex and differentiated policies and normative strategies.

In the recent decades, some processes have transformed cities in a sort of patchwork where the exposure and the encounter with the *others* are inevitable and can have an unpredictable outcome (Sandercook, 2003). Gentrification, the impoverishment of the middle class as an effect of the economic crisis, the extended form of edge cities, and above all immigration, have transformed cities creating their irregular borders in which differences, even ethnic ones, find themselves next to each other.

The increasing presence of different ethnic groups in the urban territory has led to a need for political and social reflection on the new paradigms required to interpret the city. Its kinetic character (Mehrotra, 2003) indeed develops, in a very complex direction, and requires different and innovative policies and skills necessary to govern that increasing complexity (Dioguardi, 2017). According to Myriam Grenberg the city is a conflicting space in which increasingly diversified questions are generated, starting from its being a space of plural and differentiated citizenship. Urban spaces, like larger national societies, find themselves inhabited not by abstract citizens but by individuals characterized by specificities in ethnic, cultural, and religious terms who make equally specific requests in terms of needs, projects and even desires. In the same perspective, even immigrants and each ethnic group are characterized by profound differences which policies cannot fail to take into account.

## The urban space policies

As a consequence, while cities have always been characterized by differences, they are now confronted with an unprecedented multiplicity of different cultures and ethnic groups coexisting in the same urban space. For a long time, the cities have managed the “problem of differences” through policies and practices of distancing; the designs by the prefect Haussmann in Paris, capital of the Second Empire of Napoleon III, are examples of this, as are the “two nations” that Benjamin Disraeli wrote about. Urban spaces have been designed and projected in an ethnocentric key, in order to create separation and distance to emphasize differences. But, as noted before, today, increasingly, differences coexist in the same urban space.

This new complex, fluid scenario, if not mediated by a culture of difference and inclusion, generates a widespread and undifferentiated sense of insecurity and fear (Bauman, 2005) amplified by certain narratives and political actions that create a social conviction that immigration is connected to security issues, the so-called “crimmigration” (Ambrosini, 2017). In these cities crisscrossed and often segmented by dynamic borders that render them “places with variable geometry” (Castells, 2003), managing differences becomes crucial for the same urban balances (Hollifield et al., 2014), even in the face of increasingly differentiated and widespread forms of poverty sometimes connected to a sort of spontaneous and disorganized informality^6^ (Pezzano, 2020). Cities can be either fortresses, places with enclosure walls and borders that defend those inside from those who are or should be outside, or sanctuaries that welcome and provide refuge for diversity. They fully embody the contradiction between the defense of oneself, of one's own identity and integrity and the protection of human rights and their inclusiveness (Faist, 2016; Ambrosini, 2020). The social and normative representations of these boundaries and the political choices related to these become the focal theme of cities inhabited by specific individuals with different ethnicities, cultures, and religions, and not by abstract citizens, as argued by Amin (2002) and Amin and Thrift (2002). Boundaries are invoked by those who feel they have to defend themselves from difference, from strangers (Bettini, 2020). But also by those who make their own identity a tool to claim space and recognition in the host society, which is what Ambrosini (2017) called “inversion of the stigma.”

The redesign of the urban space becomes one of the key strategic ways to shape and manage social processes (Harvey, 1989). Beyond specific policy choices, what remains confirmed is “the importance and liveliness of the urban dimension in the overall scenario of the measures aimed at governing the transformation processes in a multi-ethnic sense of the European societies”^7^ (Ambrosini, 2012). Today the city appears to be more than ever a set of fragments characterized by multiculturalism, which is often addressed in terms of integration in a “melting pot”: a homogenization of differences rather than the more advanced—but also more complex—logic of “the salad bowl” proposed by Glazer and Moynihan (1970).

This process has led to a focus on the urban policies regarding those differences, as well as on the outcomes that different choices have produced, their risks and their potential. But, at the same time, the essential starting point is the recognition of “otherness” that inhabits our cities and our daily lives.

Starting from his spatial intuition, Henri Lefebvre recognized the indissoluble link between urban space and everyday life (Lefebvre, 1974, 1976). He saw the city as a new open space where social processes take physical shape and within which it is possible to engage mechanisms of transformation. From this perspective, the role of urban space policies is fundamental. These policies can be reconducted into two different patterns: first, the spontaneous “waiting processes” of virtuous composition of differences; second, “active implement actions” to manage differences and processes of active inclusion (Amendola, 2016). These processes can also coexist in different parts of the city, which is now increasingly crisscrossed by immaterial as well as real borders to the extent that it becomes a fragmented set of urban and social spaces that do not communicate each other.

As observed, the first pattern of urban policies, the *waiting* ones, trust in spontaneous processes of integration and virtuous composition of differences. These political strategies start from the assumption that relationships, when left to develop naturally through daily dynamics, spontaneously generate conditions, and opportunities for meeting and “exposure to difference,” producing a sort of cross-fertilization of cultures.

As an alternative to the “negative policies,” that is those which involve the state and local administrations abstaining from directly intervening, there are the “positive ones.” This second pattern is based on the implementation of actions to “govern” differences, in order to prevent potential conflicts and to facilitate processes of active inclusion. Advocates of this second policy model consider these actions necessary, because of the complex dynamics of cultural change, which are unlikely to be triggered spontaneously in the absence of specific intervention policies designed to move beyond any logic of emergency governance (Verdirame and Harrell-Bond, 2005) and the pathologization of migrants.

The wait-and-see strategies, however, have already proved limited in cities, like those in North America, that have a longer history of multi-ethnic settlements, and have ended up experiencing the juxtaposition of different cultures and ethnic groups, and where still today the material and cultural signs of material and cultural separateness of those differences and of those mixes in their urban space are evident. “In the United States, the coexistence of ethnic neighbourhoods worked well for about a century. Little Italy, the Chinatowns, the Jewish Ghettos, the Harlems, the German Yorktown, where immigrants who came from distant countries could find a first settlement among people who spoke the same language, ate the same food, dressed the same way. It was thought that starting from this “mosaic” of characteristic neighbourhoods, the city could act as a melting pot or crucible, allowing integration at a later time. Then we realised that (…) discriminatory mechanisms prevailed whereby segregation triggered a vicious circle of poverty, degradation, violence and crime” (Martinotti, 2017, p. 235). The same interpretative and planning caution was suggested by Richard Sennet when, in “Flesh and stones” (Sennet, 1994), he wrote that the apparent multiculturality of Greenwich Village in New York was actually the result of the simple coexistence in a territory of cultural and ethnic groups, based on mutual “polite indifference.” These different ethnicities did not engage in dialogue, they did not take an interest in others, they did not seek each other out^8^. The groups simply coexisted in the city by exercising a sort of mutual “civil carelessness” (Albrow, 1997), occupying different portions of the urban space or, in some cases, temporally segmenting the city and occupying the same space at different times of the day (Sennet, 1990).

In any analysis of this second type of policy, it is necessary to take account of the wide variety of strategies adopted, a variety which is a reflection of an equally varied depiction of the differences and the relationship between identity and borders.

## The challenge of migrations

Within this complex scenario, problems arise when the encounter becomes inevitable, as in the new city—characterized by mixed identities and irregular borders—, which becomes a battleground between differences that have not been mediated, with a resulting exponential increase in the risk of conflict. Imposing one culture over all the others is, too often, the easiest solution to adopt. This assimilatory drive disaccustoms people from otherness, which is accepted only to the extent that it is weakened by its deepest difference; the other culture is forced to resemble the culture of the host country. Thus, we end up accentuating the difficulty of interacting with the others and their culture and implicitly consolidating the assumption that we have nothing to learn from the others, just as we have nothing to gain from contact with that diversity. This could lead either to social extraneousness and mutual indifference between different ethnic groups or to overt conflict. Today, without a culture and a politics of difference, the inevitable encounter often results in clashes and conflict, even in cities such as those in Italy where, due to a long tradition of emigration and the absence of colonies, there is no culture of immigration and integration.

An essential starting point is a policy for urban space that can lower the risks of experiences of separateness and ensure more inclusive integration processes^9^. “Reconsidering the city as an extended space, today, means rethinking the double connection between territory/society/state, reflecting on the shift in the meaning of the term “public” after a few decades in which we became aware of the fact that the society we are dealing with is increasingly characterised as a “society of differences””(Crosta, 2010, p. 50).

In order to move from educated indifference and simple tolerance to a Habermasian process of dialogue, however, at least two conditions are necessary: the first concerns public spaces, the second is related to policies for regulating the use of urban spaces. As far as the first condition is concerned, public places such as streets and squares, in which the encounter, when there is one, is completely ephemeral, are not sufficient and do not allow people to get to know others well or to establish new ties, with the consequence that too often differences become inequalities. “The distinction between differences and inequalities is not terminological, but political. The interaction between individuals with different values sheds light on the extent to which they are ready to give up or not to the same values” (Crosta, 2010, p. 50). Indeed, private spaces, which by definition are closed, basically homogeneous and preclude entry to those who are “not invited to participate,” cannot be considered a means of achieving the goals of mixed communities. These spaces include homes, clubs, and associations, which seal their borders to defend themselves from the city and its diversity, adopting as far as possible a sort of “urban policy of distinction”—“distinction” in the sense that Pierre Bourdieu gave it. The ongoing process of privatization of public spaces is a separate issue and is beyond the scope of this work (Benn and Gaus, 1983).

In this social and political scenario, one strategic tool is the (re)planning of the public urban space to allow for the development of inclusion processes, also through “urban acupuncture” (Lerner, 2003). They are targeted and widespread structural interventions that have both an architectural-urbanistic and socio-cultural nature. Immigration has long been one of the hardest and most pressing urban challenges (Cesareo, 2000, 2015) and, accordingly, the creation of physical spaces in which different individuals have the opportunity to meet for a while and initiate processes of recognition and construction of new forms of territorial communities, based on differences and “contamination,” has a key role to play.

Workplaces, schools, universities, youth centers, sports clubs, and community social centers have the characteristics that such spaces require; they can be conceived and experienced as “third spaces” (Carrera, 2022), places where contacts become inevitable and prolonged, and can be an opportunity to overcome prejudices and pre-established patterns and to remodel cultural and social multicultural relations. Third spaces are an urban experience of crossings, of going beyond borders, which can generate inclusive practices of meeting and cultural contamination. Homi Bhabha considered the process of hybridization between differences to be central, placing it, as a priority, in the “third space”, an area of negotiation of meanings and representations (Bhabha, 1991, 2001). The third space is able to destabilize defined and consolidated hierarchies and patterns; it is a place where new opportunities and new possible reconfigurations of meanings are created. Within the city and defined spaces, the third space is the place of possibilities, of uncertainty, it is the gap through which change can make its way.

Edward Soja reformulated the concept of “third space”—referring it to the spaces of symbolic representation—as one of the key categories of postmodern culture itself, a horizon of new, to some extent liminal, interstitial spaces, within which critical changes and creative responses to changes that occur in urban space are built and deconstructed (Soja, 1996, 2007). Thus, Soja seems to be of the same mind as Lefebvre in referring to a third space that can be a place of debate between different people who meet temporarily, and sometimes even associate, to generate ideas and practices that can have an impact in political terms. Public spaces and the public sphere of the city go hand in hand, insofar as the public sphere does not mean only “what people think,” but rather shared positions as a result of meeting and—even when involving disagreement- debate on specific issues (Mazzette, 2013)^10^.

This third model of space can thus be conceived with a more evident political function, which recalls the conditions for the fulfillment of the sociability mentioned by Simmel (1903)^11^. These are third physical spaces that can host both the informal and formal dimensions, in which different individuals have the opportunity to meet for an extended time and initiate processes of building territorial communities (Lefebvre, 1968). These communities are not based on common history and culture, but rather on their own differences and on a new identity project, which can assuage the feeling of being doubly foreign, an all-too-real risk for many immigrants.

## Conclusion

At the time of the first struggles of the *sans papiers* in France, Jacques Derrida argued that migrants are like a key: “external to the inside,” they can look through the keyhole at the society and the culture they would like to enter while they abandon the comfortable pocket in which they are kept. Through the metaphor of the key, Derrida depicted migrants as suspended in a limbo (the door that cannot be opened) and as afflicted by a deep, double rupture: they are foreigners twice over, in their country of origin and in the host country, they do not belong anywhere and cannot identify with either culture. Stateless, though not by choice, they are evanescent figures, whose presence in the host countries is measured in terms of mere accounting (economic benefits, resulting from the presence of a worker with no rights, and risks, linked to the presence of representatives of a “different” culture). As the French philosopher added, even though potentially the key could be a bridge, a means of connecting two otherwise closed and non-communicating spaces, in practice it becomes, in our falsely open societies, an uncomfortable sign of a cumbersome presence, evidence of permanent incapability.

In 1997 Derrida acknowledged that France, which historically had always been a country of immigration, no longer seemed to be able to welcome migrants. While sensing the discomfort of a double inadequacy, his reflections nonetheless started from an essential assumption: the emigrant–immigrant–key had reached the threshold of the door and had to be welcomed. The door had to be opened: the immigrant had made a choice and had to be helped to overcome the emigrant condition. Driven by the political necessity of the moment, Derrida ended up ignoring the other side of the coin: that of the society of origin which, having suffered the losses caused by the mass departure of its members, reacted by rejecting them, stigmatizing their absence as betrayal. Like most of the representatives of the European progressive ideology, Derrida thus ignored the doubly negative connotation of the aforementioned limbo: not only is the immigrant–emigrant not accepted in the country of origin, but she or he is also rejected by the host country and condemned to be permanently torn between two equally hostile worlds. This unequivocal reflection resulted in an undeniable mismatch, which has distinguished many of the studies and accounts of the phenomenon: the literature on immigration is as copious as the literature on emigration is inadequate, not to say inexistent. Probably, the promotion of a transcultural (rather than a multicultural or intercultural) habitus makes it possible for Western society to look at otherness no longer as a threat, but rather as a potential resource. This is to look at the migration issue “from below”: it is not a matter of ideologically avoiding evaluation of the phenomenon as an effect of the relationship between the dominant and the submissive; it is a much more modest looking at migrant as what they are, namely fellow human beings who are “in need” just like us. It is ultimately a matter of rethinking the meaning of borders: who is behind “the barbed wire”? Only “them” or us too? Are we so similar to those who, in Etty Hillesum's day, lived in comfortable villas (Hillesum, 1985)? Are not Western people, in their comfortable homes, in their comfortable democracy, still responsible for so many wars, like “them,” behind the barbed wire? We need to redefine borders as “places of living” (Galantino, 2019), places of encounter with otherness and, ultimately, places where people can rediscover their deepest humanity.

## Author contributions

PC contributed to the sections Introduction, Us and them: The sense of boundaries, the complexity of a total social fact, and Near and far: From the reflection on immigration to the reflection on migration. LC contributed to the sections cities as places of identity and cultural conflict, the urban space policies, and the challenge of migrations. All authors contributed to the article and approved the submitted version.

## Conflict of interest

The authors declare that the research was conducted in the absence of any commercial or financial relationships that could be construed as a potential conflict of interest.

## Publisher's note

All claims expressed in this article are solely those of the authors and do not necessarily represent those of their affiliated organizations, or those of the publisher, the editors and the reviewers. Any product that may be evaluated in this article, or claim that may be made by its manufacturer, is not guaranteed or endorsed by the publisher.

## Footnotes

^1^The notion of métissage as a cultural process was established in anthropology and linguistics even before it emerged in sociology, but soon became a transdisciplinary term: the studies that most contributed to its diffusion are often from a liminal area between anthropology, philosophy, and sociology of culture. In this context, the notion of métissage has been transformed into a sort of paradigm, that is, into a new way of considering the problems posed today by international migration, globalization, and increasingly frequent inter-ethnic contacts. Literature worthy of note on this topic includes (Amselle, 1990; Nouss, 1997/2006; Callari Galli, 2005).

^2^World Migration Report 2022, International Organization for Migration.

^3^With regard to the topic of Itinerant Communities see Escofier (2009).

^4^The problem of “re-cognizing” and “re-elaborating” is addressed by Sayad (2002). The Algerian sociologist, who never became a naturalised French citizen, starts by pointing out that he himself also experienced the violence of a state that considers immigrants as intruders (or “clandestine,” to use a term a favored by the media) and demands continuous proofs of their loyalty.

^5^“The transnational anthropology” of landscapes and flows by Appadurai, in line with the work of other cultural critics such as Homi Bhabha, Edward Said, and Gayatri Spivak, (...) reconfigure the debate on globalisation not as a simple dichotomy of homologation-differentiation, but as multiple scales of hybridization, places, and people more involved in the tensions brought by cultural homologation and the simultaneous processes of differentiation, which could be called glocalization or transnationalism (Soja, 2007, p. 244).

^6^Brillembourg (2020) writes about the “non-codes” of abusive cities that follow unwritten rules of self-organization.

^7^Urban governments are the main actors of immigration policies (Pennix et al., 2004); it is at this level that conflicts arise or integration policies are activated. The gap between declared policies and policies that are actually implemented can be a problem (Campomori, 2008), an aspect that involves the propagandist-demagogic dimension of politics, which is often focused on responding to the fears and values of the electorate.

^8^In California, Margaret Crawford studied the Ethnoburbs, ethnically homogeneous micro cities—e.g., Vietnamese—living in the urban galaxy of Los Angeles or San Diego.

^9^Integration often focuses on a process of assimilation of new cultures into that of the host country, and there is a tendency to forget that the term refers to a more complex and, above all, two-way process, in which the culture of the host country also changes as a result of contact with new ones. On the other hand, inclusion processes, recalling the salad bowl metaphor, aim to welcome differences, preserving their specificities without assimilating them. This is related to the aforementioned process of overcoming the paradigm of “foreigner” (Cotesta, 2002).

^10^The “representativeness” of the public sphere derives from openness to all and from the plurality of groups and/or points of view (Sebastiani, 2007, p. 97; Mazzette, 2010, pp. 45–60).

^11^In the metropolis he wrote about, which is beyond and more than a physical place, namely an ideal type of modernity itself, sociability is based on “forms of reciprocity between individuals, protracted over time and transformed into stable, self-sufficient formations and provided with a well-defined physiognomy”.

## References

[B1] Albrow M. (1997). The Global Age. Cambridge: Polity Press.

[B2] Ambrosini M. (2012). Governare Città Plurali. Politiche Locali di Integrazione per gli Immigrati in Europa. Milano: Franco Angeli.

[B3] Ambrosini M. (2017). Migrazioni. Milano: Egea.

[B4] Ambrosini M. (2020). L'invasione Immaginaria. L'immigrazione Oltre i Luoghi Comuni. Bari: Laterza.

[B5] Ambrosini M. Abbatecola E. (2009). Migrazioni e Società. Milano: Franco Angeli.

[B6] Amendola G. (2016). Le Retoriche Della Città: Tra Politica, Marketing e Diritti. Napoli: Liguori.

[B7] Amin A. (2002). Ethnicity and the multicultural city: living with diversity. Environ. Plann. A 34, 959–980. 10.1068/a3537

[B8] Amin A. Thrift N. (2002). Città. Ripensare la Dimensione Urbana. Bologna: il Mulino.

[B9] Amselle J.-L. (1990). Logiques Métisses. Anthropologie de L'identité en Afrique et Ailleurs. Paris: Payot.

[B10] Appadurai (1996). Modernity at Large: Cultural Dimensions of Globalization, Minneapolis, MN: University of Minnesota Press.

[B11] Barrett M. (2013). Interculturalism and Multiculturalism: Similarities and Differences. Strasbourg: Council of Europe Publishing.

[B12] Bauman Z. (2005). Fiducia e Paura Nella Città. Milano: Bruno Mondadori.

[B13] Beck U. Giddens A. Lash S. (1994). Reflexive Modernization. Cambridge: Polity Press.

[B14] Benn S. Gaus G. (1983). Public and Private in Social Life. London: Cromm Helm.

[B15] Bettini M. (2020). Hai sbagliato foresta. Il furore dell'identita. Bologna: ilMulino.

[B16] Bhabha H. (1991). “A question of survival: Nations and psychic states,” in Psychoanalysis and Cultural Theory: Thresholds, ed J. Donald (St. Martin's Press), 89–103.

[B17] Bhabha H. (2001). I Luoghi Della Cultura. Roma: Meltemi.

[B18] Bourdieu P. (1984). Questions de Sociologie. Paris: Les Éditions De Minuit.

[B19] Bourdieu P. (1998). Meditazioni Pascaliane. Milano: Feltrinelli.

[B20] Brillembourg A. (2020). “Caracas: città ineguale,” in Nove (Ri)Tratti Urbani per un Viaggio Planetario, P. Piscitelli, Atlante delle città (Torino: Feltrinelli), 203–231.

[B21] Burgess E. W. (1925). “The growth of the city,” in The City, eds R. Park, E. W. Burgess, R. D. McKenzie (Chicago), 47–62.

[B22] Callari Galli M. (2005). Il Meticciato Culturale. Luogo di Creazione di Nuove Identità o di Conflitto? Milano: Cluem.

[B23] Cambi F. (2006). Incontro e Dialogo: Prospettive Della Pedagogia Interculturale, Roma, Carocci.

[B24] Campomori F. (2008). Immigrazione e Cittadinanza Locale. La Governance Dell'integrazione in Italia. Roma: Carocci.

[B25] Carrera L. (2022). Designing inclusive urban places. Ital. Sociol. Rev. 12, 141–158. 10.13136/isr.v12i1.52236078581

[B26] Castells M. (2002). La Nascita Della Società in Rete. Milano: Egea.

[B27] Castells M. (2003). Il Potere Delle Identità. Milano: Egea.

[B28] Cesareo V. (2000). Società Multietniche e Multiculturalismi. Milano: Vita e Pensiero.

[B29] Cesareo V. (2015). La Sfida Delle Migrazioni. Milano: Vita e Pensiero.

[B30] Cotesta V. (2002). Lo Straniero. Pluralismo Culturale e Immagini dell'Altro Nella Società Globale. Roma-Bari: Laterza.

[B31] Crosta P. (2010). Pratiche. Il Territorio è “L'uso Che Se Ne Fa”. Milano: FrancoAngeli.

[B32] De Sousa Santos B. (2011). “Introducción: las epistemologías del sur,” in Formas–Otras. Saber, Nombrar, Narrar, Hacer, CIDOB (Org.) (Barcelona: CIDOB Ediciones), 9–22.

[B33] Della Porta D. Greco M. Szakolezai A. (eds.). (2000). Identità, Riconoscimento, Scambio. Laterza: Roma-Bari.

[B34] Demetrio D. (2003). “Implicazioni interculturali nella ricerca dell'interiorità,” in Pedagogia Interculturale in Italia e in Europa. Scritti in onore di Luigi Secco, ed A. Portera (Milano: Vita e Pensiero).

[B35] Dioguardi G. (2017). Per una Scienza Nuova del Governo della Città. Roma: Donzelli Editore.

[B36] Escofier C. (2009). “Transmigration et communautés d'itinérances au Maghreb,” in Le Maghreb à L'épreuve des Migrations Subsahariennes: Immigration sur Emigration, ed A. Bensaad (a cura di) (Paris: Karthala Editions).

[B37] Faist T. (2016). Cross border migration and social inequalities. Annu. Rev. Sociol. 42, 323–346. 10.1146/annurev-soc-081715-074302

[B38] Galantino N. (2019). Sul Confine. Milano: Piemme.

[B39] Glazer N. Moynihan D. P. (1970). Beyond the Melting Pot: The Negroes, Puerto Ricans, Jews, Italians, and Irish of New York City. Cambridge: Cambridge University Press.

[B40] Hannerz U. (1996). Transnational Connections. London: Routledge.

[B41] Harvey D. (1989). The Condition of Postmodernity. Blakwell: Oxford.

[B42] Hifly M. A. Berthomiere W. Mihaylova D. (2004). La notion de rosea reseaux sociaux en migration. Hommes Migrat. 1250, 6–12. 10.3406/homig.2004.4206

[B43] Hillesum E. (1985). Diario 1941–1943. Milano: Adelphi.

[B44] Hollifield J. F. Martin P. L. Orrenius P. M. (eds.). (2014). Controlling Immigration: Global Perspective. Stanford, CA: Stanford University Press.

[B45] Jedlowski P. (2000). Storie Comuni: La Narrazione Nella Vita Quotidiana. Turin: Pearson Italy.

[B46] Laplantine F. Nouss A. (1997). Le Métissage. Paris: Flammarion. (It. Transl. by Il Pensiero Meticcio, Milan: Elèuthera, 2006).

[B47] Le Quéau P. (2007). L'homme en Clair-Obscur: Lecture de Michel Maffesoli, Saint-Nicolas. Québec, QC: Les Presses de l'Université de Laval.

[B48] Lefebvre H. (1968). Il Diritto Alla Città. Bologna: Il Mulino.

[B49] Lefebvre H. (1974). La Produzione Dello Spazio. Milano: Moizzi.

[B50] Lefebvre H. (1976). Spazio e Politica. Milano: Moizzi.

[B51] Lerner J. (2003), Acupuntura Urbana. Rio de Janeiro: Iaac.

[B52] Martinotti F. (2017). Sei Lezioni Sull'urbanistica. Milano: Franco Angeli.

[B53] Mazzette A. (a cura di). (2010). Pratiche sociali di città pubblica. Bari: Laterza.

[B54] Mazzette A. (a cura di.). (2013). Pratiche Sociali di Città Pubblica. Roma-Bari: Laterza.

[B55] Mehrotra R. (2003). “Static spaces, kinetic places. public space in mega city of Bombay,” in Cities and Market Conference, IFHP World Congress (Vienna).

[B56] Melucci A. (2000). “Costruzione di sé, narrazione, riconoscimento,” in Identità, Riconoscimento, Scambio, eds D. Della Porta, M. Greco, and A. Szakolezai (Roma-Bari: Laterza).

[B57] Nouss A. 1997 (2006) XXV Rapporto Immigrazione, Il pensiero meticcio.

[B58] Ortiz F. (1940). Contrapunteo Cubano del Tabaco y el Azúcar, J. Montero, Habana, 67, It. Transl. *Contrappunto del Tabacco e Dello Zucchero*, Introduzione di B. Malinowski (1982). Milano: Rizzoli.

[B59] Pennix R. Kraal K. Martinello M. Vertovec S. (2004). Citizenship in European Cities. Immigrants, Local Politics and Integration Policies. Aldershot: Ashgate Publishing Lmt.

[B60] Pezzano A. (2020). “Johannesburg: città frammentata,” in Atlante Delle Città. Nove (Ri)Tratti Urbani per un Viaggio Planetario, ed P. Piscitelli (Torino: Feltrinelli), 31–48.

[B61] Pratt M. L. (1992). Imperial Eyes: Travel Writing and transculturation, London: Routledge.

[B62] Pratt M. L. (1995). Reworking Modernity: Capitalisms and Symbolic Discontent. New Brunswick, NJ: Rutgers University Press.

[B63] Rea A. Tripier M. (2003). Sociologie de L'immigration. Paris: La Découverte.

[B64] Sandercook L. (2003). Cosmopolis II: Mongrel Cities of the 21st Century. London: A&C.

[B65] Sayad A. (1976). L'immigration Algérienne en France. Paris: Entente.

[B66] Sayad A. (2002). La Doppia Assenza: Dalle Illusioni Dell'emigrato Alle Sofferenze Dell'immigrato. Cortina: Milano.

[B67] Schulze-Engler F. (2002). “Transnationale Kultur als Herausforderung für die Literaturwissenschaft,” in Zeitschrift für Anglistik und Amerikanistik, A Quarterly of Language, Literature and Culture, eds M. Butter, L. Eckstein, J. Frenk, B. Georgi-Findlay, T. Herbst, B. Korte, G, Leypoldt, C. Reinfandt, and A. Stefanowitsch (Berlin: De Gruyter).

[B68] Sebastiani C. (2007). La politica delle città, Il Mulino. Bologna.

[B69] Secchi B. (2013). La Città dei Ricchi e la Città Dei Poveri. Roma-Bari: Laterza.

[B70] Sennet R. (1990). The Coscience of Eye: The Design and Social Life of Cities. New York, NY: Knopf.

[B71] Sennet R. (1994). Flesh and Stones: The Body and the City in Western Civilization. New York, NY: Norton and Company.

[B72] Simmel G. (1903). La Metropoli e la Vita Dello Spirito. Roma: Armando Editore.

[B73] Soja E. W. (1996). Thirdspace: Journey to Los Angeles and Other Real-and-Imaginated Places. Malden: Blackwell.

[B74] Soja E. W. (2007). Dopo la Metropoli: Per Una Critica Della Geografia Urbana e Regionale. Bologna: Patron.

[B75] Tarsia T. (2010). Aver Cura del Conflitto: Migrazioni e Professionalità Sociali Oltre i Confini del Welfare. Milano: Franco Angeli.

[B76] Verdirame G. Harrell-Bond B. (2005). Rights in Exile: Janus-Faced Humanitarianism. New York, NY: Berghahn Books. 10.2307/j.ctt1x76fpq

[B77] Welsch W. (1999). “Transculturality: the puzzling form of cultures today,” in Spaces of Culture: City, Nation, World, eds M. Featherstone and S. Lash (London: SAGE), 194–213. 10.4135/9781446218723.n11

